# Chronic heart failure treatment benefits from pro-BNP-directed therapy

**Published:** 2011

**Authors:** Aalbers J

**Affiliations:** Special Assignments Editor

The PROTECT study,^1^ which was undertaken to evaluate the benefits of tailoring heart failure therapy according to NT-proBNP levels, was stopped early at the midpoint of the trial due to the early benefits of this targeted biomarker approach. Initially, the study planned to recruit 300 patients with New York Heart Association (NYHA) class II–IV systolic heart failure [left ventricular ejection fraction (LVEF) ≤ 40%] but was stopped after only 151 patients were recruited.

All the PROTECT patients received standard-of-care heart failure therapy, with clinically guided up-titration of medication, while 75 patients had their therapy adjusted to drive levels of aminoterminal pro-B-type natriuretic peptide (NT-proBNP) down to the pre-specified target of ≤ 1 000 pg/ml.

Nearly half of the patients in the guidedtherapy arm actually achieved the target, and as a group they showed a significant reduction in the primary endpoint (p = 0.019) over a mean of 10 months, compared with standard-of-care patients. Their hazard ratio for the composite of total cardiovascular events (worsening heart failure, hospitalisation for heart failure, acute coronary syndrome, ventricular arrhythmias, cerebral ischaemia, or cardiovascular death) was 0.44 (95% CI: 0.22–0.84) after adjustment for age, LVEF, NYHA class and renal function.^2^

The results of the PROTECT trial were presented at the recent American Heart Association (AHA) 2010 congress. While not yet published in a scientific journal, fairly detailed results were released at the meeting.

In his presentation at the AHA, Dr James Januzzi of the Massachusetts General Hospital noted that changes in NT-proBNP were strongly associated with the presence and severity of heart failure and were markedly related to progression of heart failure. NT-proBNP levels also dropped in response to therapy. ‘However, prior trials of NT-proBNP-guided therapy have delivered mixed results. Also the elderly have not responded as well as younger patients’, he noted.

While patients in the NT-proBNP arm were seen more frequently at the heart failure clinic in order to drive their biomarker levels down, up-titration of drug therapy occurred in both the standard-of-care and NP-proBNP arms, showing that patients in the standard-ofcare arm also received aggressive therapy. Interestingly, differences in therapies occurred in the NT-proBNP-guided arm, with a significantly increased use of aldosterone antagonists and lower use of loop diuretics. Forty-four per cent of patients achieved the NT-proBNP target level of less than 1 000 pg/ml.

In the NT-proBNP arm, a significant reduction in total cardiovascular events occurred (a drop of 50%), mainly driven by a reduction in worsening heart failure and heart failure hospitalisation. Primary and secondary outcomes are summarised in Table 1.

**Table 1:** PRIMARY AND SECONDARY OUTCOMES IN PROTECT

*Parameter*	*NT-proBNP guided, n = 75*	*Standard of care only, n = 76*	p*-value*
NT-proBNP levels (pg/ml)			
Baseline	2344	1946	NS
10-month follow up	1125^1^	1844	0.03
Primary endpoint^2^ events (n)	58	100	0.009
Patients with a primary event (%)	29.3	43.4	0.04
Quality of life,^3^ rate of improvement by 10 points (%)	61.2	38.8	0.03
			

^1^*p *= 0.01 vs baseline.

^2^Primary endpoint = total cardiovascular events (worsening heart failure, hospitalisation for heart failure, acute coronary syndrome, ventricular arrhythmias (VT/VF), cerebral ischaemia, or cardiovascular death.

^3^As measured by the Minnesota Living with Heart Failure questionnaire.

Therapies in both arms were well tolerated and importantly, patients older than 75 years of age showed similar benefits. There was however an excess of hypotension in the NT-proBNP arm. The measured quality-of-life (QOL) parameters improved in both arms, but a greater improvement occurred in the NT-proBNP arm.

In conclusion, Dr Januzzi pointed out that the PROTECT results have shown a very robust outcome and the benefits of this guided approach, ‘if duplicated in larger trials, indicate that NT-proBNPguided therapy may well represent a paradigm shift in the treatment of the heart failure patient’.
